# Supplementary figures and images for: Anti-Leptospira immunoglobulin profiling in mice reveals strain specific IgG and persistent IgM responses associated with virulence and renal colonization
Source: PLoS Negl Trop Dis. 2021 Mar 11;15(3):e0008970. doi: 10.1371/journal.pntd.0008970 (PMC8007020; doi:10.1371/journal.pntd.0008970)

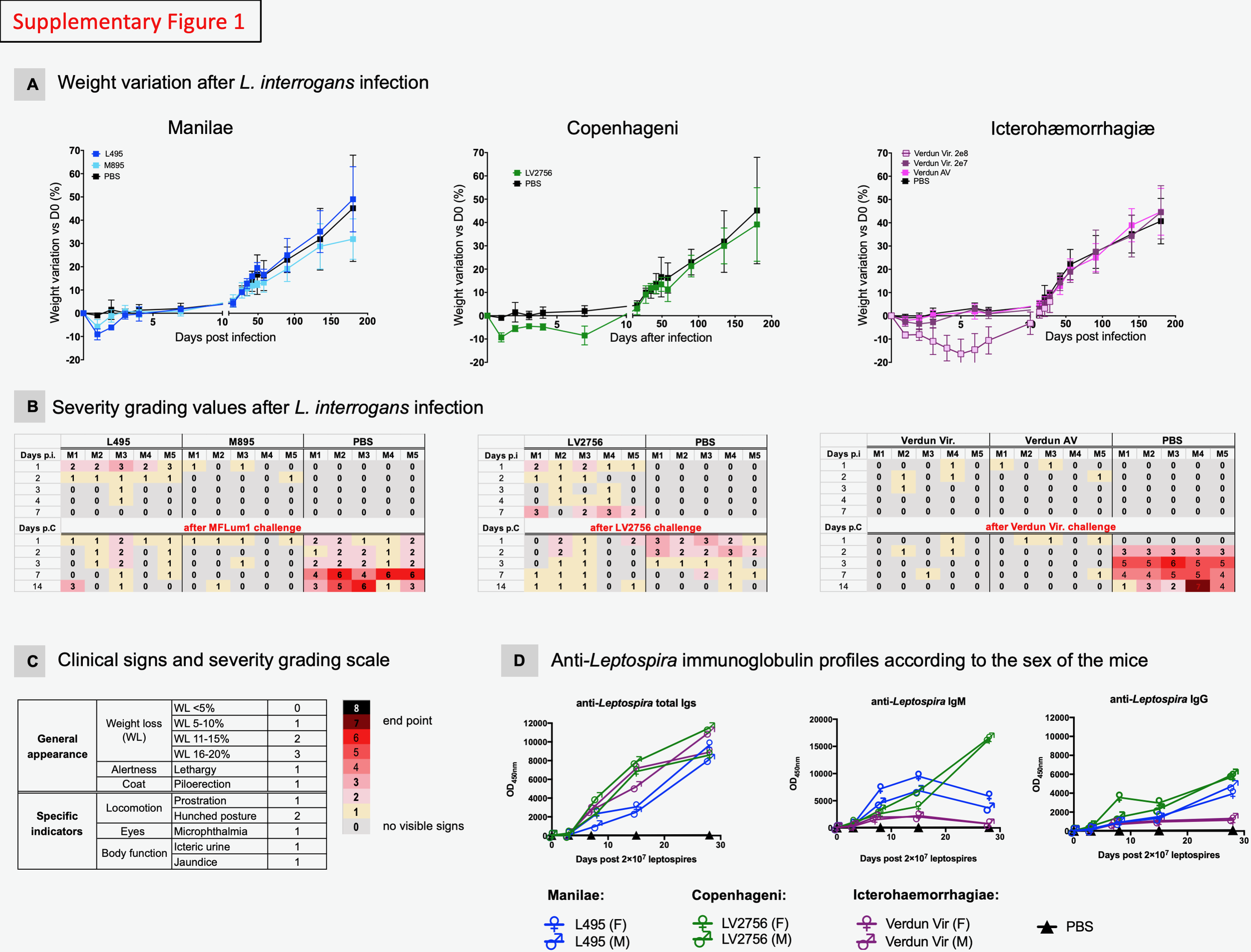

Supplement: S1 Fig — Outcome evaluation after pre-infection or subsequent infection in C57BL/6 mice with virulent strains or avirulent mutants representative of 3 distinct pathogenic (Manilae, Copenhageni, Icterohaemorrhagiae) serovars. A) Weight evolution individually recorded (n = 5 mice per group) in mice inoculated with 2×10^7^ of virulent Manilae L495 strain or M895 mutant, or virulent Copenhageni Fiocruz LV2756 strain, or virulent Icterohaemorrhagiae Verdun (2 doses) or avirulent (AV) strains, or PBS as negative control. Weight changes from the initial weight at the day of infection, expressed as a percentage, was daily recorded from D0 to D7 p.i., then weekly (D8 to the end of the experiment). Graphs represent the mean ± SD of the weight change recorded overtime for each experimental group. B) Colored score values individually recorded at defined time-points after infection (Days p.i.) as in A) and after homologous challenge (Days p.C.). n = 5 mice /group, identified M1 to M5 in each group. C) List of clinical signs recorded after infection with Leptospira to establish individual severity scores as indicated in the color code. A score value equal to or greater than 7 out of 11 defines an end point limit. D) Profiles of specific Ig (left panel), IgM (central panel) and IgG (right panel) for 1 month in male or female mice (n = 5/group), after experimental infection with 2×10^7^ virulent strains of Manilae, Copenhageni and Icterohaemorrhagiae serovars, or PBS as negative control. (TIF) [file pntd.0008970.s001.tif]

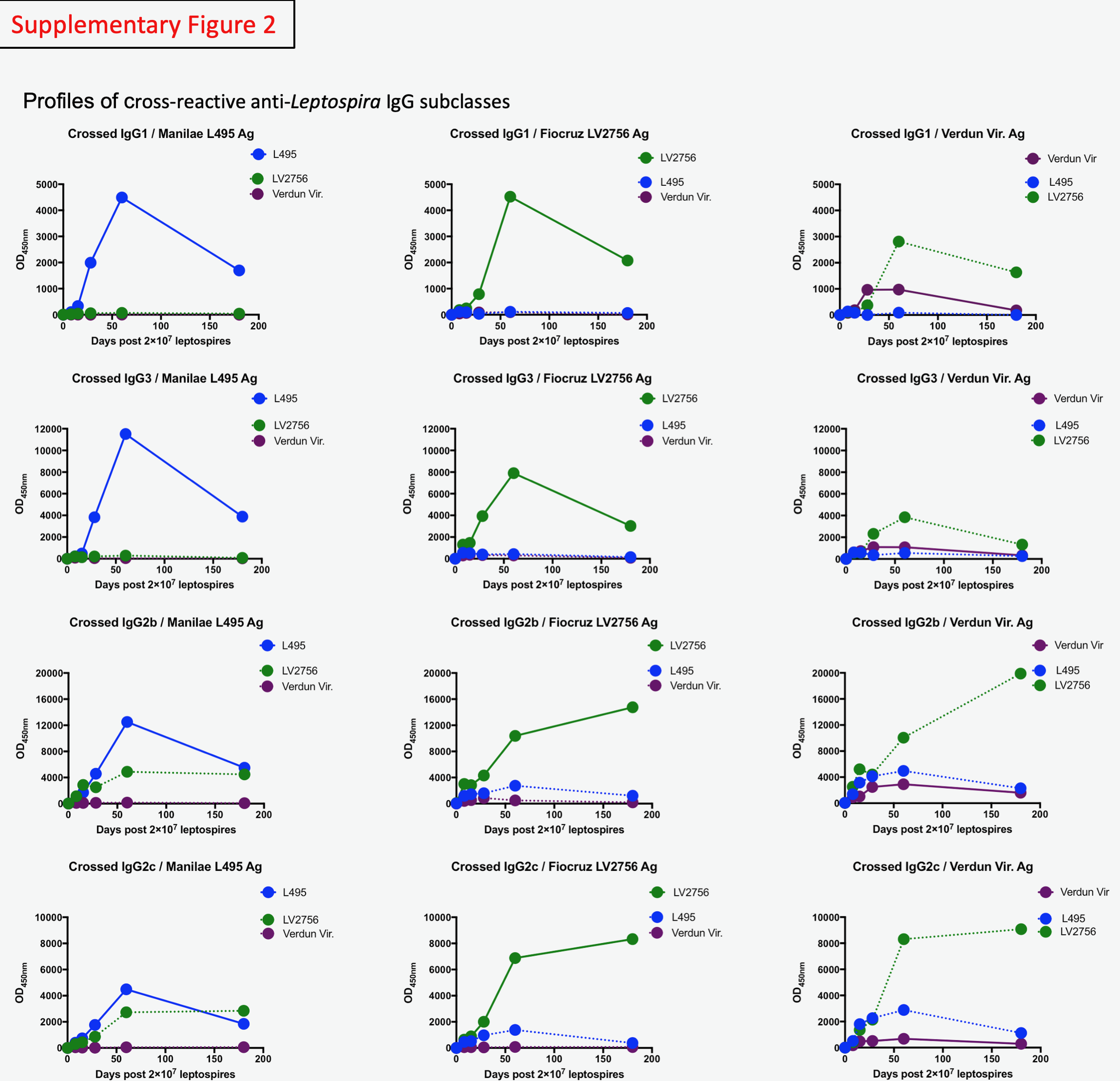

Supplement: S2 Fig — Profiles of different IgG subclasses of anti-Leptospira immunoglobulins tested against antigens from heterologous serovars. Specific IgG1 (first row), IgG3 (second row), IgG2b (third row) and IgG2c (bottom/fourth row) immunoglobulins obtained after experimental infection with 2×10^7^ virulent leptospires representative of 3 distinct serovars, Manilae L495 (left column), or Copenhageni Fiocruz LV2756 (central column) or Icterohaemorrhagiae Verdun (right column), were checked against heterologous serovar antigen preparation (dashed line) and compared to the profile obtained with homologous serovar (full line). Anti-Leptospira IgG subclasses were determined by ELISA assay using specific Leptospira antigen preparations and appropriate dilutions of serum collected at determined time-point after infection. Each figure represents the profiles for specific IgG1, IgG3, IgG2b and IgG2c subclasses obtained from serum tested in pool (for a same experimental group). Antibody responses were assessed up to day 180 p.i. with female mice (n = 5). (TIF) [file pntd.0008970.s002.tif]

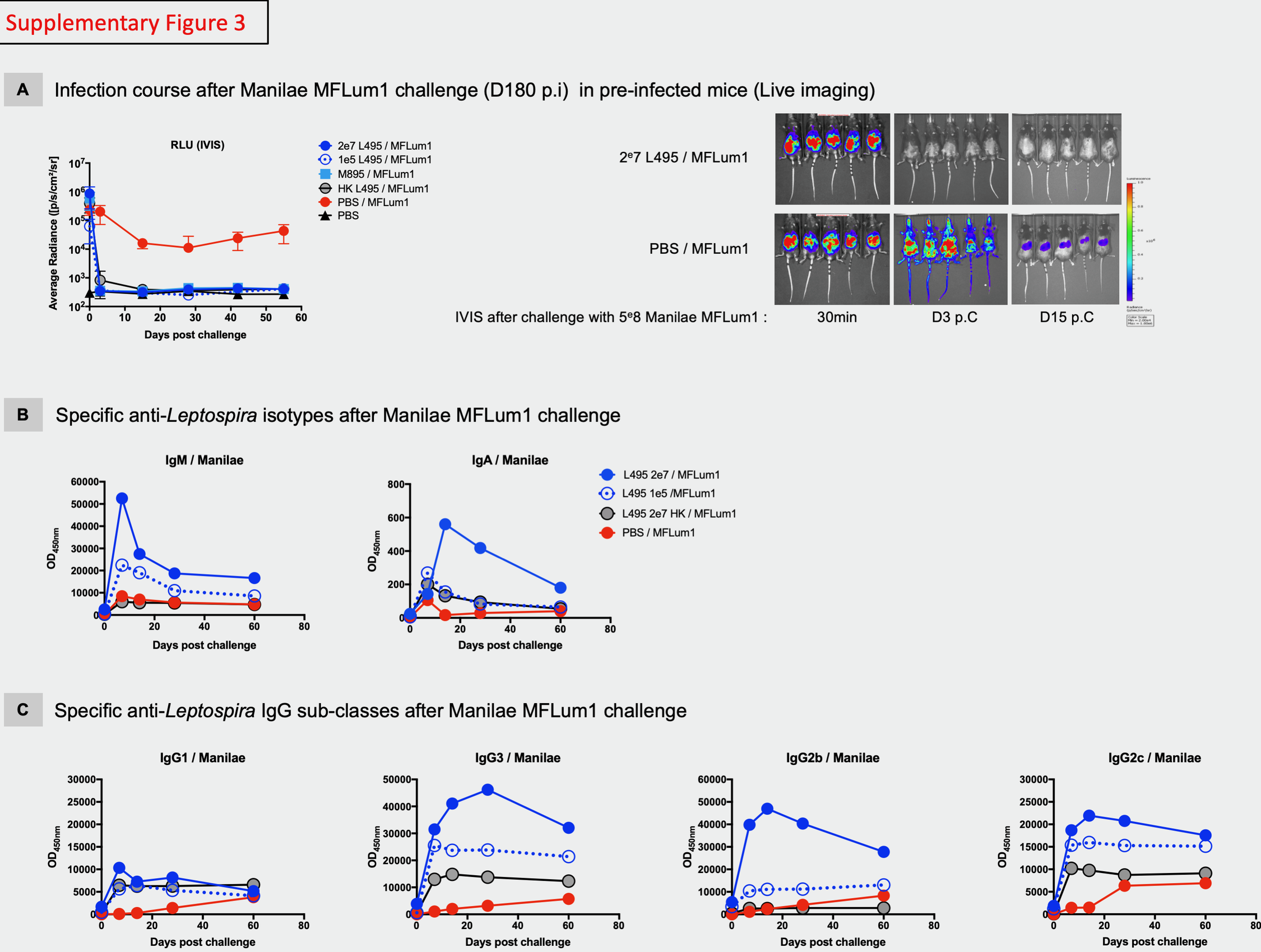

Supplement: S3 Fig — Outcome monitoring and specific Ig responses in C57BL/6J mice intraperitoneally pre-infected with different doses of avirulent or inactivated Manilae L495 and, after 6 months, challenged with the Manilae bioluminescent derivative MFLum1 strain. A) Relative light units (RLU) measured by live imaging (IVIS) in mice pre-infected with 2×10^7^ or 1×10^5^ of virulent L495 strain or 2×10^7^ of M895 mutant or 2×10^7^ of heat-killed L495 or PBS and challenged with 5×10^8^ of MFLum1 strain (left panel). The IVIS has been performed as recently described [102], at 30 min, D3, D15, D28 and D55 post-challenge (p.C.). The graph represents the mean ± SEM of the average radiance in n = 5 mice in each group, imaged in ventral (D0 and D3 p.C.) then in dorsal position, and gated on the whole body. The background level of light was measured on control C57BL/6 mice injected with PBS at the time of infection (PBS). Representative images of 2×10^7^ Manilae L495 or PBS pre-infected mice challenged with MFLum1 and then, tracked by IVIS at 30 min, D3 and D15 post-challenge (p.C.) (right panel) with n = 5 mice /group. The blue to red scale is proportional to the intensity of bioluminescence, reflecting the number of live leptospires. B) Profiles of specific anti-Leptospira isotype (IgM, IgA) and C) IgG subclass responses (IgG1, IgG3, IgG2b, IgG2c) produced in mice inoculated with 2×10^7^ (blue line) or 1×10^5^ (dashed blue line) of alive virulent L495 strain or 2×10^7^ (grey line) of heat-killed L495 or PBS as control, then challenged with 5×10^8^ MFLum1. Specific Ig responses were determined by ELISA immunoassay using specific Leptospira antigen preparation and appropriate dilutions of serum collected at defined time-point after challenge. Each figure represents the profiles for each type of Ig obtained from serum tested in pool (for a same experimental group with n = 5 mice /group). Antibody responses were assessed up to day 60 p.i. (TIF) [file pntd.0008970.s003.tif]
