# Supplementary material for: Anti-Leptospira immunoglobulin profiling in mice reveals strain specific IgG and persistent IgM responses associated with virulence and renal colonization
Source: PLoS Negl Trop Dis. 2021 Mar 11;15(3):e0008970. doi: 10.1371/journal.pntd.0008970 (PMC8007020; doi:10.1371/journal.pntd.0008970)
Supplement: Attachment — Submitted filename: 2021_02_12 point by point answer FVP humoral.docx [file pntd.0008970.s004.docx]

We thank the reviewers for their positive comments and suggestions. We accordingly revised the manuscript and all changes are highlighted in yellow.

Here are the point-by-point answers to the reviewers’ questions.

**Answers to Reviewer #1:**

Lines 191 and 421: to conform with standard nomenclature for animal experimentation, it is recommended that the word “killed” be replaced with “euthanized”.

Thank you, this has been done.

Figure 3A: it is recommended that the Y axis be expanded to decrease the clustering of the data points, and to increase the ability to view these data points, especially at the early time periods.

Thank you, this has been done and now corresponds to the figure 4A.

Lines 336-338 and discussion: to understand the significance of the differing response to the infectious dose, the authors should indicate, if known, the expected dose during a natural infection.

This is an interesting point difficult to address since no one knows the dose, kinetics and the route of natural infections (transdermal, mucosal). Moreover, it may be different from one animal to another since the resistance of animals to leptospirosis is highly variable.

However, using 16S rRNA qPCR, a few studies have tried to quantify concentrations of leptospires and detected in soil from Brazilian slums around 4.16×102 GEq/g [1] and in water around 152 GEq/mL [2], suggesting that the dose we used in our “resistant” mouse model is indeed very high compared to what is found in environment. In our study, we also tested a lower dose, that usually does not induce renal colonization, but we cannot infer whether it may mimic more closely what would happen upon natural infection in mice.

Figure 3B, left panel: -why did two previously infected mice have a higher leptospiral organ burden?  Was there a different immune response detected in these two outlier mice.

The two mice exhibiting renal colonization correspond to mice injected with a low dose (10^5^ leptospires) and subsequently challenged with a high dose of leptospires (5x10^8^). Our explanation for this renal colonization is that the first infection did not trigger a potent enough Ig response compared to the high dose (shown in figure 1B), and 2 mice out of 5 get slightly colonized after the challenge infection. We could hypothesize that the protection conferred by the first infection is dose dependent. This is now reformulated in the result section lines 452-454.

Whether a different immune response occurred in these two mice (S4 and S5) is an interesting point. Therefore, we characterized over 6 months the Ig profiles in individual sera from mice injected with the low dose (S1 to S5 in the graphs below). No differences in IgM or total IgG could be observed. We have some tendencies in the IgG1 and perhaps IgG3 sub-classes but it is not possible to drive conclusions from 2 mice out of 5. There was no possibility to repeat this experiment in the frame of the present study and therefore we did not add nor discussed these results.

There appears to be a disconnect between the findings shown in Figure 1A and Figure 3A, in that the avirulent serovars provided a minimal humoral response but still protected the mice from subsequent homologous infection.  On Line 647-650 the authors offer an explanation for a similar result obtained with heat-killed Manilae; do the authors expect cell-mediated immunity also plays a role in the observed immunity with the avirulent serovars?

Whether cellular immunity can play a role in the defense of mice is an interesting and open question that we aim to investigate next. In this regard, we added a new paragraph (line 696-702) about what is known about cellular immunity, mostly in cattle.

However, if the avirulent serovar elicited low levels of antibodies after the first infection, the antibody response was strikingly boosted upon challenge, suggesting that indeed the adaptive humoral immunity is functional, and could potentially provide by itself the protection.

**Answers to Reviewer #2:**

Since the main focus of the study is measuring immunoglobulin types/subtypes, more information is needed on the ELISA used in this study, eg., (i) was this assay developed/validated in-house?  Please provide a reference(s) in either case.

The ELISA assay was indeed developed for this study and validated in-house and optimized to detect immunoglobulins recognizing carbohydrates but also protein antigens in their native conformation. The mat/met section has been modified accordingly. This also largely corresponds to the protocol used in the recent published article from the Wunder/Ko’s group showing the ability of the attenuated Fiocruz f*cpA* mutant to act as a live cross-reactive vaccine through immunoglobulins directed at protein antigens [3].

(ii) is there any known cross-reactivity among different anti-mouse antibodies used in this study?

Yes, we agree that this is an important control that was performed. We added in the mat/met section line 237: *“In each experiment, non-specific reactivity of HRP-labelled specific anti-mouse Ig was checked on leptospiral antigens. OD values were all in the range of 0,05-0,07 considered as the background of the assay. Data were expressed as mean values of duplicates or triplicates, subtracted from the background value”.*

2. Is it possible that the observed relationship between persistent peripheral IgM levels and renal colonization has something to do with the immune environment of kidneys and this phenomenon has some similarity to immune deviation seen during infection of sites like brain, eyes, testes etc.?

This is an interesting question. Our hypotheses developed in the discussion line 571 could potentially be true if leptospires could settle and colonize in these immune protected sites from the blood circulation, such as the brain and eye. In the latter, it is unlikely since the uveitis observed in horses is not due to the presence of persistent leptospires, but it is thought to be immune mediated and would originate from cross-reactive antibodies directed against *Leptospira* Lru proteins that mimic the host proteins.

**Answers to Reviewer #3:**

-The limitations of the study could be better communicated.

As raised also by reviewer 1, we added 2 paragraphs to discuss the relevance and limitations of our mouse model regarding the route and dose of infection.

1) We added in the introduction, line 136, a paragraph; *“Recent studies in mice with L. interrrogans Copenhageni Fiocruz L1-130 showed that experimental transdermal infection or through ocular and nasal mucosa, which are considered as natural routes of infection, lead, like the classical intraperitoneal injection, to blood dissemination and renal colonization, although with different kinetics. In addition, all these routes lead 15 days post infection to comparable levels of total specific leptospires immunoglobulins. Therefore, in this work, using our established mouse model of intraperitoneal infection, we…*

2) The limits of our study concern the use of mice that are resistant to leptospirosis, and thus different from humans who are susceptible to develop an acute life-threatening disease. Therefore, we modified the conclusion paragraph line 723: *“Although our study concerns mice that are resistant animal to leptospirosis, and thus different from humans who are susceptible to develop an acute life-threatening disease, the mouse model will be useful to address the mechanisms linked to long-term protection and potentially understand why there is not such a long-lasting protection in human leptospirosis”.*

We thank the reviewers for the discussion and nice suggestions that we hope have improved the manuscript.

In addition to the reviewers’s comments, we sought that testing the cross-reactivity of the sera elicited toward one serovar against the other antigens was missing in our study. Therefore, we performed the experiment and added a figure (new Figure 3) and new paragraphs in the result and discussions sections with references.

-In the result section lines 387-406: we added a paragraph:

***“Cross-reactive anti-Leptospira immunoglobulins in sera of pre-infected mice***

*Humoral immunity against Leptospira following infection is mainly described as LPS-dependent and consequently as serovar-specific. However, cross-reactions against related serovars have been reported [3, 4]. We assessed the cross-reactivity of anti-Leptospira immunoglobulins obtained after pre-infection with the virulent strain of three distinct serovars against the two other serovars’antigens.*

*While specific immunoglobulins obtained after Manilae L495 or Verdun infection did not show strong evidence of reactivity with the other serovars, we unexpectedly observed that IgM, IgA and IgG antibodies resulting from the Copenhageni Fiocruz LV2756 infection cross-reacted strongly with Icterohaemorrhagiae Verdun antigen and IgG, with Manilae L495 antigen (Figure 3). Accordingly,all IgG four subclasses elicited after Fiocruz infection strongly cross-reacted and recognized Verdun antigens, and to a lesser extent Manilae antigens. Interestingly, while IgG1 and IgG3 elicited by Manilae infection barely cross-reacted, IgG2 (b and c) against Manilae cross-reacted well with both Verdun and Fiocruz antigens, although the Ig subclasses elicited after Verdun infection did not cross-react with Fiocruz, nor with Manilae (Sup Figure 2).*

*These results show that the low homologous humoral responses observed after the Verdun infection are not due to a technical issue in antigen preparation, and rather suggest a specific ability of the Icterohaemorrhagiae Verdun strain to avoid the development of a strong immunoglobulin response. In addition, the results also suggest the existence of a complex immune response after Leptospira infection with some serovars able to confer cross-reactivity. Interestingly, we evidenced that the cross-reactivity conferred by one serovar against another is not necessarily a reciprocal relationship”.*

-In the discussion section, line 537, we added a sentence to explain that the cross-reactivity towards the Verdun’s antigen provides an internal control to justifies the fact that the lack of homologous recognition of the Verdun’s antigen is not artifactual:

*“the results about cross-reactivities showing that other serovars can recognize Verdun antigens strongly suggest the specific capacity of Verdun virulent strain to dampen the specific humoral response.”*

-line 647; we added the following paragraph;

“*Cross-reactivity experiments showed that infection with the Fiocruz strain triggered cross-reactive antibodies against both Manilae L495 and Icterohaemorrhagiae Verdun strains. This is in line with the recent study showing that the attenuated live Fiocruz fcpA mutant confers cross-protection against infection with other serovars, including Manilae [3]. Here, we show that the Fiocruz strain triggers high amounts of IgG1 and IgG3, which by large recognize proteins. However, if these IgG1 and IgG3 cross-reacted with the Icterohaemorrhagiae serovar, they do not cross-react with Manilae, although in contrast, IgG2 elicited with Fiocruz cross-reacted with both Manilae and Verdun. These results are difficult to reconcile with the data showing that the antigenic cross-protection of fcpA was due to antibodies directed against highly conserved proteins of leptospires, rather than neutralizing antibodies directed at serovar specific LPS [3].*

1.	Schneider AG, Casanovas-Massana A, Hacker KP, Wunder EA, Jr., Begon M, Reis MG, et al. Quantification of pathogenic *Leptospira* in the soils of a Brazilian urban slum. PLoS Negl Trop Dis. 2018;12(4):e0006415. Epub 2018/04/07. doi: 10.1371/journal.pntd.0006415. PubMed PMID: 29624576; PubMed Central PMCID: PMCPMC5906024.

2.	Casanovas-Massana A, Costa F, Riediger IN, Cunha M, de Oliveira D, Mota DC, et al. Spatial and temporal dynamics of pathogenic *Leptospira* in surface waters from the urban slum environment. Water Res. 2018;130:176-84. Epub 2017/12/09. doi: 10.1016/j.watres.2017.11.068. PubMed PMID: 29220718; PubMed Central PMCID: PMCPMC5767135.

3.	Wunder EA, Adhikarla H, Hamond C, Owers Bonner KA, Liang L, Rodrigues CB, et al. A live attenuated-vaccine model confers cross-protective immunity against different species of the *Leptospira* genus. Elife. 2021;10. Epub 2021/01/27. doi: 10.7554/eLife.64166. PubMed PMID: 33496263; PubMed Central PMCID: PMCPMC7837694.

4.	Sonrier C, Branger C, Michel V, Ruvoen-Clouet N, Ganiere JP, Andre-Fontaine G. Evidence of cross-protection within *Leptospira interrogans* in an experimental model. Vaccine. 2000;19(1):86-94. Epub 2000/08/05. doi: 10.1016/s0264-410x(00)00129-8. PubMed PMID: 10924790.
